# Accuracy and Timeliness of Knowledge Dissemination on COVID-19 Among People in Rural and Remote Regions of China at the Early Stage of Outbreak

**DOI:** 10.3389/fpubh.2021.554038

**Published:** 2022-01-11

**Authors:** Wen Zhou, Leshui He, Xuanhua Nie, Taoketaohu Wuri, Jinhai Piao, Dunshan Chen, Hui Gao, Jianmin Liu, Kyedrub Tubden, Ming He, Jun He

**Affiliations:** ^1^School of Ethnology and Sociology, Yunnan University, Kunming, China; ^2^Department of Economics, Bates College, Lewiston, ME, United States; ^3^College of Ethnology and Anthropology, Inner Mongolia Normal University, Hohhot, China; ^4^Institute of Ethnic Studies, Yanbian University, Yanbian, China; ^5^Center for Collaborative Innovation in the Heritage and Development of Xizang Culture, Xizang Minzu University, Xianyang, China; ^6^School of Political Science and Laws, Shihezi University, Shihezi, China; ^7^College of Ethnology and Sociology, Guangxi University for Nationalities, Nanning, China; ^8^Tibet's Economic and Cultural Research Center, Tibet University, Lhasa, China

**Keywords:** infectious diseases, COVID-19, communication inequality, risk communication, disadvantage groups

## Abstract

Coronavirus disease 2019 (COVID-19) spread throughout China in January 2020. To contain the virus outbreak, the Chinese government took extraordinary measures in terms of public policy, wherein accurate and timely dissemination of information plays a crucial role. Despite all of the efforts toward studying this health emergency, little is known about the effectiveness of public policies that support health communication during such a crisis to disseminate knowledge for self-protection. Particularly, we focus on the accuracy and timeliness of knowledge dissemination on COVID-19 among people in remote regions—a topic largely omitted in existing research. In February 2020, at the early-stages of the COVID-19 outbreak, a questionnaire survey was carried out. In total, 8,520 participants from seven less economically developed provinces situated in the borderlands of China with large ethnic minority groups responded. We analyzed the data through poisson regression and logistic regression analyses. We found that (1) people in remote regions of China obtained accurate information on COVID-19. Further, they were able to take appropriate measures to protect themselves. (2) Result from both descriptive analysis and multivariable regression analysis revealed that there is no large difference in the accuracy of information among groups. (3) Older, less educated, and rural respondents received information with a significant delay, whereas highly educated, younger, urban residents and those who obtained information through online media were more likely to have received the news of the outbreak sooner and to be up to date on the information. This research provides evidence that disadvantage people in remote regions obtained accurate and essential information required to act in an appropriate manner in responses to the COVID-19 outbreak. However, they obtained knowledge on COVID-19 at a slower pace than other people; thus, further improvement in the timely dissemination of information among disadvantage people in remote regions is warranted.

## Introduction

In the early 2020s, China encountered a serious public health emergency after coronavirus disease 2019 (COVID-19) was first diagnosed in Wuhan in December 2019 (1) and subsequently spread throughout the country in less than a month. In late February, cases of COVID-19 were confirmed in every province of China, including remote areas and mountainous regions. Only two month of COVID-19 outbreak, official news reported 80,924 confirmed cases, with 3,140 deaths in China (2). Since the person-to-person transmission of COVID-19 was scientifically confirmed (3, 4), the Chinese government has taken tremendous efforts to inform the public and control further transmission. These measures include putting cities on lockdown, restricting public transportation, limiting migration of labor and traveling, closing stores and other business operations, mandating the use of face masks in public, as well as various other measures of quarantine (5). The effectiveness of such measures, however, largely depends on public awareness on the risk and people's knowledge regarding self-protection. As such, it is a great challenge to ensure that scientific information is broadly received. In particular, it is additionally challenging to deliver information and knowledge regarding the virus outbreak to marginalized people in a timely manner, including ethnic minorities who are more likely to live in remote areas and suffer from poverty, thus potentially being more vulnerable to the risk of the disease because of the lack of local medical support and healthcare resources.

Globally, rapid reaction to the outbreak of infectious diseases is limited by the dissemination of accurate information among the public in a timely manner (6, 7). Existing research suggests that a better understanding of the health risks can lead to improved self-protection, which can significantly support transmission control, as in the case of SARS (8, 9), MERS (10), and Ebola (11, 12). The accessibility and quality of information disseminated during a public health emergency may vary substantially across groups with different socioeconomic statuses – a communication inequality was documented during public health emergencies like the H1N1 outbreak (13, 14), hurricane Katrina (15) and the Zika virus outbreak (16). The literature also highlights additional social factors underlying health inequalities that are associated with socioeconomic backgrounds, including ethnicity, poverty, education level, and geographic constrains (17–21). This study contributes to the literature with a timely and quantitative analysis during the current COVID-19 outbreak to evaluate whether marginalized people receive accurate and timely information, and in doing so, provides support for evidence-base policy making.

Since the outbreak of COVID-19, there has been a rapid growth in the literature on COVID-19, with studies predominantly conducted by medical and epidemiological scientists. Most current research focuses on modeling and predicting the potential population that might be infected (22, 23). Others explore the mechanisms of transmission (24). Numerous studies have also examined the originality and molecular structure of the virus (25). Whereas scientific research has improved the understanding of the transmission and control of COVID-19, few studies from social sciences have examined the effects of information communication on transmission control, particular among people in remote regions.

To fill this gap, we carry out a large-scale survey focusing on the effects of health communication during the early stages of the COVID-19 outbreak in the beginning of February 2020. This is a critical point of time when the Chinese government officially implemented drastic measures under a state of emergency to contain the outbreak and formally disseminated information regarding the virus and self-protection. As such, this research aims to make three empirical contributions, as follows: (1) to contribute as one of first evidence focusing on the socioeconomic aspects of COVID-19 transmission control; (2) to provide evidence regarding communication inequality in COVID-19; (3) to provide information on the implications of timely policy implementation in China and other countries on the containment of COVID-19 and other related infectious diseases.

## Methods

### Data Collection

This research focuses on the borderland area of China during the COVID-19 outbreak. We conducted our survey in seven different provinces of China—Guangxi, Yunnan, Tibet, Xinjiang, Inner Mongolia, Heilongjiang and Jilin—all of which are international-boarder provinces of China and are home to a significant proportion of the ethnic minority population. The results of the 6th census in 2010 showed that the ethnic minority populations in these 7 provinces account for 50.28% of the non-Han majority group in China. Most of these provinces are landlocked and less economically developed regions of China. National statistics have shown that these provinces ranked among the bottom 12 of the 31 provinces^1^ in China by annual total GDP in 2018.

The survey was undertaken from the 5 February to 9 February 2020, the booming stage of the COVID-19 outbreak in China, following the World Bank survey approach applied for an Ebola study (26) and most updating COVID-19 studying (27). This survey was carried out through a questionnaire survey platform, wherein respondents participated online with smartphones to avoid physical contact among individuals. Through the cooperation of 7 universities in each of these 7 provinces, we used snowball sampling to collect our responses, which initially started through social networks of research teams in each university and later spread through social media networks to more respondents.

Because we aimed to collect timely responses during a fast-evolving health emergency across a diverse set of geographical areas while overcoming travel bans and quarantine, we were unable to organize a standard random sampling process. Despite this shortcoming, we made several efforts to reach a wide geographic coverage, while striving for representativeness. First, to ensure representation of respondents from a diverse set of regions in the sample, we relied on our existing field work networks to recruit respondents from targeted communities. Specifically, we chose 10 counties from each of the 7 provinces and two villages or communities from each county as targets. In each of these 20 villages^2^, one local investigator directly contacted the local village and community leaders via telephone to ensure that at least 15 respondents participated. These steps provided more coverage of non-urban residents in our sample (41.59%, see Appendix Table 1). Second, we explicitly encouraged our students from the 7 universities to spread our questionnaire to their senior relatives, family members, and residents in the same community, to mitigate the overrepresentation of university students in the sample (a sample average age of 32 years and 37.36% of participants >35 years; see Appendix Table 1). Third, to improve the efficacy, we excluded responses that were completed too fast or too slowly. Considering a baseline average completion time of 6 min during our pre-test, we excluded all responses that were completed under 3 min or over 50 min.

As a result, our effective sample included a total of 8,520 respondents (see Appendix Table 1 for the descriptive statistics of our data), with 66.88% being female and 41.15% belonging to ethnic minorities. The average age of our sample was 32.43 years, with 46.63% between 21 and 35 years old and 37.36% older than 35 years. Of the participants, 29.53% were rural residents; 11.12%, suburban residents; and 59.35%, urban residents—slightly higher than the average urbanization rate of 7 provinces in 2019 (54.4%). The average completion time for the questionnaire was approximately 8 min (mean = 469.07 seconds, S.D. = 300.97). All thought we made several efforts to reach a non-urban and senior residents, Yunnan province is over-represented because we want to make the sample reach to more ethnic minority residents, urban and younger residents are a little bit over-represented in our sample (see Appendix Table 2).We analyze and evaluate these limitations in the last section of the article.

### Measurement

Gaps exist in information dissemination, particularly health-related information, during a state of emergency. One type of gap is the difference between what people need to know and what they already know, another type is the delay between when people need to know and when people actually receive information. We refer to the former type of gap as *information gap in accuracy* and the latter, as *information gap in timeliness*.

For information gap in accuracy, three indicators were developed: (1) accuracy of facts, which tested whether people were able to correctly identify rumors and scientifically reliable information about COVID-19. In the questionnaire, we listed 6 COVID-19-related statements, among which 4 statements were rumors and the other 2 were scientifically confirmed facts. The respondent was asked to check whether each statement was true or false, and the respondents received the full score of 6 if they correctly distinguished all of them. (2) Accuracy of methods tested whether the respondent knew of the effective preventative measures advocated by health professionals. In the questionnaire, we use a multiple choices question asking what could be done to prevent infection. The options of this question included 4 effective measures and other ineffective confounders. If the respondent chose all four right answers, they receive full marks of 4 for this measure. (3) Accuracy of action tested whether a respondent had taken effective advocated measures to protect himself/herself. We used a multiple-choice question asking what measures the respondent had taken after they had learned about the COVID-19 outbreak. Five of the options were advocated effective measures, and the respondent received 1 point for each effective measure chosen, with the full score being 5.

To measure the information gap in timeliness, two indicators were developed: (1) early-known stands for whether people received information about the outbreak before 20 January 2020, which is the date on which person-to-person transmission of COVID-19 was officially confirmed and a state of public health emergency was declared nationally. (2) Newly known is an indictor to examine whether people learned the newly discovered transmission route of COVID-19, which is a fecal-oral path of transmission^3^, although it still needs more research to confirm. We used this indicator to measure whether the respondent was able to obtain up to date information in a timely manner—an essential feature in the fast-changing environment of a health emergency.

Existing research shows that mass media plays a key role in health communication (7, 31). In particular, compared with traditional media, online media may respond to news more quickly (8); however, it disproportionately serves younger, more highly educated, and urban demographics, thus leading to a communication inequality (32). To test the effect of media type to information gap, we distinguished two type of media. If respondents get the outbreak news at first through the newspaper, TV, or neighbors and friends, we define them as first informed by traditional media, if respondents get the outbreak news at first through WeChat, Weblog or online news App, we define them as first informed by online media.

### Data Analysis

First, we cross-tabulated our measures of information gap by group. Next, we presented evidence from poisson regression models and ordered logit regression models that measure the size and significance of information accuracy gap across different groups, while controlling for province fixed effects, self-evaluated health level, and whether the respondent knew of any confirmed and suspected cases nearby. Next, we used logit model to estimate the information gap in timeliness between different groups. In addition, we used mediation analysis to measure the intermediary effect of online media on information gap in timeliness.

## Results

### Information Gap in Accuracy

Table 1 presents summary statistics of the information gap in accuracy by sex, age group, educational background, rural-urban residence, and ethnicity. The means of all three indicators were found to be very close to the full scores. For example, the mean of accuracy in action is 4.7 out of a full score of 5, implying that most respondents took all 5 appropriate measures to protect themselves. These preliminary comparisons suggest that the information gap in accuracy is very small.

**Table 1:** Information gap in accuracy among groups.

		**Accuracy of facts**		**Accuracy of methods**		**Accuracy of action**	
		**(full score = 6)**		**(full score = 4)**		**(full score = 5)**	
		**Mean**	**Test**	**Mean**	**Test**	**Mean**	**Test**
Sex	Female	5.13	*F =* 3.22	3.91	*F =* 54.30	4.69	*F =* 3.22
	Male	5.09	(*p =* 0.07)	3.82	(*p =* 0.00)	4.52	(*p =* 0.07)
Age	10 to 20	5.01	*F =* 10.72	3.89	*F =* 1.44	4.60	*F =* 2.95
	21 to 35	5.14	(*p =* 0.00)	3.89	(*p =* 0.21)	4.62	(*p =* 0.02)
	36 to 50	5.14		3.88		4.71	
	51 to 65	5.01		3.84		4.61	
	>65	5.04		3.82		4.59	
Education	Primary school	4.31	*F =* 109.75	3.60	*F =* 20.86	4.29	*F =* 7.12
	Middle school	4.69	(*p =* 0.00)	3.78	(*p =* 0.00)	4.61	(*p =* 0.00)
	High school	4.81		3.86		4.69	
	College	5.14		3.89		4.65	
	Post-graduate	5.34		3.90		4.59	
Residential	Rural	4.91	*F =* 113.38	3.84	*F =* 19.28	4.55	*F =* 29.40
type	Suburban	5.14	(*p =* 0.00)	3.90	(*p =* 0.00)	4.67	(*p =* 0.00)
	Urban	5.23		3.90		4.67	
Ethnicity	Minority	4.87	*F =* 129.30	3.84	*F =* 11.71	4.60	*F =* 6.13
	Han-Chinese	5.21	(*p =* 0.00)	3.90	(*p =* 0.00)	4.64	(*p =* 0.01)

*The test in the table is multiple-comparison test by analysis-of-variance (ANOVA) models. The numbers in parentheses are p values for the significant test*.

On analyzing the data by group, we found that the difference across groups by sex, education attainment, residential type, and ethnicity was clear for all three indicators—female, highly educated individuals, urban residents, and Han-Chinese respondents report more accurate information. Most of them are statistic significant by multiple-comparison test except the difference between female and male. These observations indicate that, during the COVID-19 outbreak, highly educated individuals, urban residents, and Han-Chinese residents in China were more likely to have better information on protecting themselves and taking appropriate actions. The difference across age groups is less clear. Overall, participants of the age group 36–50 years report more accurate information than those of other age groups.

Most of the differences we discussed above are quite small. For example, the accuracy of the methods score is 3.89 for participants aged 10–20 years and 3.82 for those aged >65 years, a difference of only 0.07 points. Similarly, the difference is only 0.06 points between rural and urban residences, ethnic minorities, and Han-Chinese participants. The greatest gap in estimates observed was between different education groups. The accuracy of fact scores for the primary-school-educated group was 4.31, whereas that for the post-graduate-educated group was 5.34—a sizable difference of 0.97 or 1 standard deviation. The accuracy of the methods score between these two groups was 0.3 or approximately 0.4 standard deviations of the measure.

We use a multivariate linear regression model and an ordered logit model to measure and test the information gap in accuracy between groups. The three indicators of accuracy were the independent variables in each model. Our key explanatory variables were age group, sex, educational attainment, rural-urban residential type, and ethnicity. Our main control variables were self-evaluated health status and province indicators because health status affect people's reaction to virus and different province get different situation of virus infection and control policies We also controlled for three additional variables on potential exposure to the virus to control for the underlying variation in respondents' local environment that could have intensified their interests and thus may have affected information accuracy, including whether the respondent knows of any suspected or confirmed cases in the family or among friends or classmates (SC-RFC), in the same village or community (SC-SV), or in the neighboring village or community (SC-NV).

Table 2 reports estimates from the poisson regression models and ordered logit models, which are largely consistent with each other, showing the robustness of our results. Overall, these more precise estimates echo our previous observations—the differences across most groups are statistically significant. The respondents who are older, female, better educated and living in urban and sub-urban area were significantly better informed across two or all three measures. Although ethnicity considered to be a dominant feature of borderland area of China, the information gap between ethnic minority and ethnic Han is quite small and two of the three estimates are not statistically significant. It means Han-Chinese respondents report more accurate information we show in table 1 may because they have different residential type and education attainment.

**Table 2:** Multivariate regression analysis of the information gap in accuracy among groups.

	**Accuracy of facts**		**Accuracy of methods**		**Accuracy of action**	
	**Poisson**	**Ologit**	**Poisson**	**Ologit**	**Poisson**	**Ologit**
Age	−0.001	−0.005	0.003^**^	0.031^**^	0.003^***^	0.056^***^
	(0.001)	(0.020)	(0.001)	(0.010)	(0.001)	(0.013)
Age squared	0.000	−0.000	−0.000^**^	−0.000^**^	−0.000^**^	−0.001^***^
	(0.000)	(0.000)	(0.000)	(0.000)	(0.000)	(0.000)
Sex (female = 0)	−0.014^***^	−0.519^***^	0.007	0.096^*^	−0.031^***^	−0.425^***^
	(0.003)	(0.087)	(0.005)	(0.044)	(0.004)	(0.058)
Education (primary = 0)						
Middle school	0.016	0.294	0.019	0.143	0.029	0.421^*^
	(0.015)	(0.243)	(0.021)	(0.159)	(0.018)	(0.200)
High school	0.031^*^	0.537^*^	0.036	0.272	0.040^*^	0.530^**^
	(0.015)	(0.238)	(0.020)	(0.153)	(0.017)	(0.191)
College	0.039^**^	0.866^***^	0.096^***^	0.776^***^	0.037^*^	0.456^*^
	(0.014)	(0.225)	(0.019)	(0.146)	(0.017)	(0.179)
Postgraduate	0.034^*^	0.706^**^	0.129^***^	1.152^***^	0.017	0.066
	(0.014)	(0.242)	(0.020)	(0.152)	(0.017)	(0.187)
Ethnic minority	−0.004	−0.165	−0.022^***^	−0.220^***^	−0.003	−0.112
	(0.002)	(0.093)	(0.004)	(0.044)	(0.004)	(0.060)
Area (rural=0)						
Suburban	0.011^**^	0.341^*^	0.036^***^	0.348^***^	0.020^***^	0.306^**^
	(0.004)	(0.154)	(0.008)	(0.073)	(0.006)	(0.098)
Urban	0.014^***^	0.448^***^	0.037^***^	0.357^***^	0.025^***^	0.387^***^
	(0.003)	(0.108)	(0.006)	(0.052)	(0.005)	(0.070)
Observations	8520	8520	8520	8520	8520	8520
Log likelihood	−15638.07	−10516.05	−14009.01	−2598.37	−15049.64	−5772.79

* *p < 0.05*,

** *p < 0.01*,

*** *p < 0.001*.

*Coefficients (log odds from ordered logit models and log count from Poisson models) have been presented. The numbers in parentheses are standard errors. All regressions control for provinces fixed effects; self-reported health statues; and SC-RFC, SC-SV, and SC-NV. Their estimates are omitted here but are available upon request*.

Although many estimates are statistically significant, the coefficients are relatively small, which means the information accuracy gap between groups are quite small. For example, compared with respondents in the primary school educated group, respondents in Postgraduate educated group get 3.4% increase (e^0.034^-1) in the count of Accuracy of Facts. Compared with rural residents, the count of Accuracy of Facts for the urban residents increased by 1.4% (e^0.014^-1) and for the sub-urban residents, by 1.1% (e^0.011^-1).

The small information gap in accuracy shows the comprehensive coverage of the virus outbreak in China since late January, with effective communication of essential information to a wide group of diverse residents in areas far from the epicenter of the outbreak. The evidence supporting the effective transmission of information is consistent with the slowdown of transmission in areas outside of the Hubei province in China since mid-February. We argue that the success in informing the public is instrumental in slowing the spread of the virus abroad. As the Director-General of the World Health Organization (WHO), Dr. Tedros Adhanom Ghebreyesus, noted on 15 February 2020^4^: China has taken strong public health measures and efficiently utilized its resources to respond and manage the outbreak and spread of COVID-19 since mid-January. Propaganda machinery has been running on full steam to inform the public. National news has been covering daily press conferences by the government and educational videos, folk songs, banners, advertisements in buses and on billboards, etc. on COVID-19 have been used to inform and motivate people to isolate themselves and fight against the outbreak. All residents have been encouraged to work from home and stay indoors, and companies and shops have been encouraged to suspend business.

**Figure 1:**
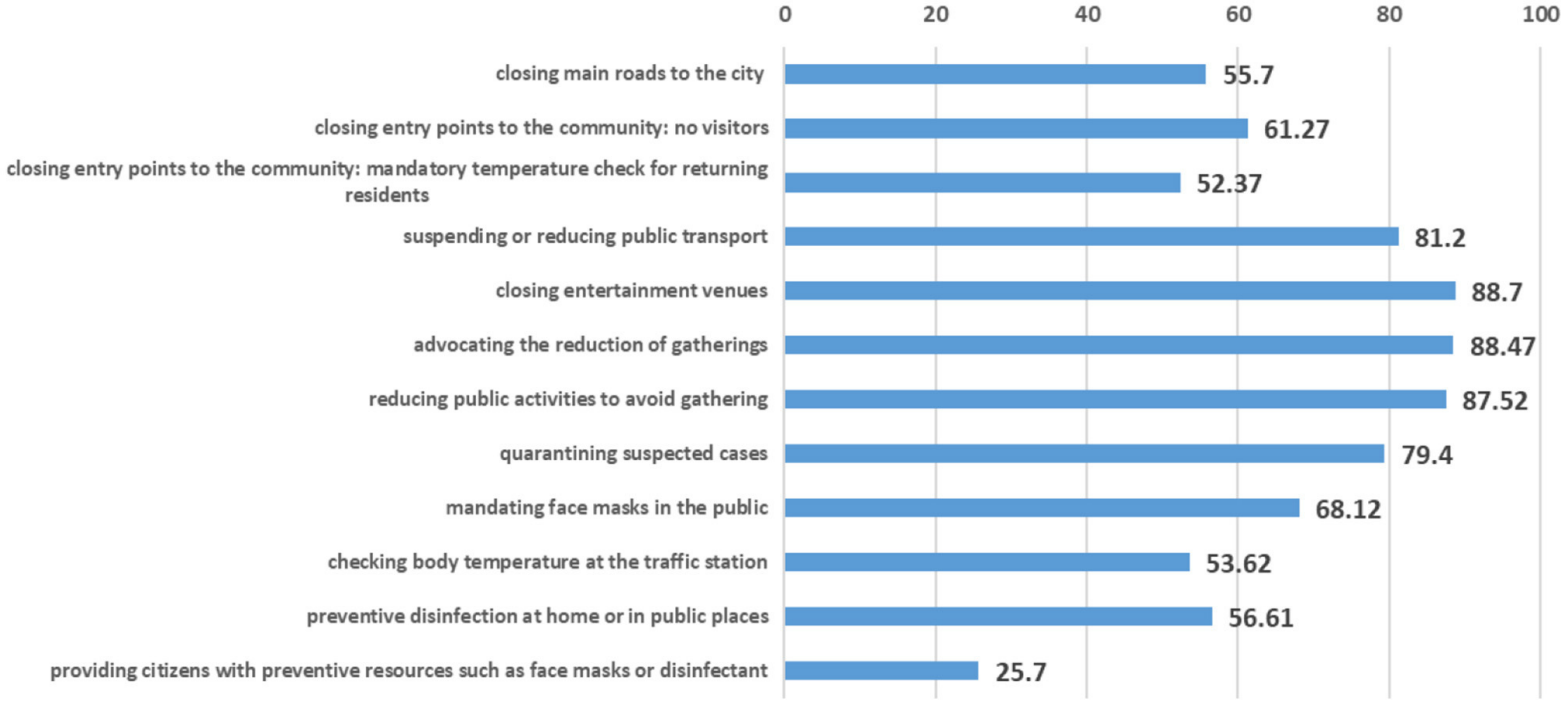
Percentage of resident communities or local governments that adopted various prevention and control measures to fight COVID-19.

An effective information campaign is conducive to the marked quarantine measures that have disrupted the way of life among most Chinese people and have had a significant financial impact. Since late January, dramatic measures, such as school cancelation, store closure, and limited public transportation, have been imposed across China to contain the virus. Our survey also attempted to collect information on the extent to which such measures were taken by local communities and governments during the outbreak. In our sample, more than 98.82% of the respondents' communities or local governments had taken measures to stop the spread of the virus. Although the 7 broader provinces are far from Wuhan, more than 85% of the respondents reported that local communities had taken measures, such as closing entertainment venues, persuading residences to reduce gatherings, and reducing public activity to avoid gatherings. More than 79% of the respondents reported that public transportation facilities had been suspended or reduced and that suspected cases had been quarantined. More than 50% of the respondents reported that the main roads into or out of their cities and access routes to the villages or communities had been closed, preventive disinfection had been performed at home or in public areas, and that their temperature had been measured at bus and train stations. About 24.38% of the respondents reported that the residential communities or local government provided preventive resources, such as face masks and disinfectant, to the residents (see Figure 1).

### Information Gap in Timeliness

The elderly group and the low-educated group had a large information gap in timeliness compared with the other groups. Table 3 (column 1) shows the proportion of early-known respondents by group. The differences were substantial, especially across different age group, educational attainment levels and across the rural-urban divide. The fraction of early-known in the 10–20-year group was 79.77%, but it was only 49.12% among those older than 65 years. Among the primary-school-educated group the fraction of early-know was 55.95%, whereas in the post-graduate-educated group, it was 68.33%.

**Table 3:** Information gap in timeliness among groups.

		**Early-known**		**Newly known**	
		**Rate**	**Chi^2^ test**	**Rate**	**Chi^2^ test**
Sex	Male	67.87	*x^2^* = 3.15	63.6	*x^2^* = 66.97
	Female	65.95	(*p =* 0.07)	54.39	(*p =* 0.00)
Age	10–20	79.77	*x^2^* = 241.22	53.96	*x^2^* = 45.68
	21–35	70.35	(*p =* 0.00)	61.11	(*p =* 0.00)
	36–50	58.36		64.31	
	51–65	57.27		56.38	
	>65	49.12		66.67	
Education	Primary school	55.95	*x^2^* = 105.94	39.88	*x^2^* = 83.87
	Middle school	55.14	(*p =* 0.00)	52.78	(*p =* 0.00)
	High school	58.86		56.17	
	College	70.44		60.79	
	Post-graduate	68.33		66.89	
Residential type	Rural	68.84	*x^2^* = 5.43	52.42	*x^2^* = 98.76
	Suburban	68.11	(*p =* 0.05)	63.67	(*p =* 0.00)
	Urban	66.26		64.01	
Ethnicity	Han-Chinese	66.19	*x^2^* = 5.93	63.72	*x^2^* = 51.26
	Minority	68.71	(*p =* 0.01)	56.02	(*p =* 0.00)

*The numbers in column 1 and column 3 are the proportion of early-known and the proportion of newly-known. The numbers in parentheses are p values for the significant test*.

Further, Table 3 (column 2) also shows that younger, more educated, and urban respondents were much more likely to be informed of the updated route—showing a sizable gap in the latency of information updates across groups.

We use a logit regression model to estimate the variations in these measures attributable to social demography variables (Table 4). Column 1 shows a significant negative association between age and the likelihood of early-known. Our estimates show that a one-year increase in age was associated with an 11.13% decrease (1-e^−0.118^) in the odds of early-known. The coefficient of educational attainment was significantly positive for the college-educated and post-graduate-educated groups, compared with the primary-school-educated group. The odds of early-known for the college-educated group increased by 40.63% (e^0.341^-1) and for the post-graduated-group, by 54.34% (e^0.434^-1).Compared with rural residents, urban residents had significant advantages in terms of early-known. Compared to the obvious difference across groups by age, education attainment and residential type, the coefficient of ethnicity is not significant, which shows again that ethnicity may not be a major factor in risk information inequality.

**Table 4:** Regression analysis of the information gap in timeliness among groups.

	**Early-known**	**Newly known**	**Media type**	**Early-known**	**Newly known**
	**(1)**	**(2)**	**(3)**	**(4)**	**(5)**
Age	−0.118^***^	0.002	−0.123^***^	−0.022^***^	0.004
	(0.012)	(0.011)	(0.004)	(0.002)	(0.002)
Sex (female = 0)	0.072	−0.347^***^	−0.867^***^	0.112^**^	−0.332^***^
	(0.051)	(0.049)	(0.071)	(0.052)	(0.049)
Education (primary = 0)					
Middle school	−0.017	0.205	1.556^**^	−0.024	0.227
	(0.185)	(0.186)	(0.760)	(0.184)	(0.185)
High school	0.049	0.293	1.745^**^	0.092	0.282
	(0.179)	(0.179)	(0.748)	(0.177)	(0.178)
College	0.341^*^	0.473^**^	2.756^***^	0.338^**^	0.462^***^
	(0.172)	(0.172)	(0.740)	(0.170)	(0.171)
Postgraduate	0.434^*^	0.562^**^	3.075^***^	0.331^*^	0.624^***^
	(0.178)	(0.178)	(0.743)	(0.177)	(0.177)
Ethnic minority	0.011	−0.150^**^	0.063	0.006	−0.149^***^
	(0.052)	(0.049)	(0.062)	(0.052)	(0.049)
Area (rural = 0)					
Suburban	0.144	0.315^***^	0.199^**^	0.105	0.327^***^
	(0.087)	(0.082)	(0.101)	(0.087)	(0.081)
Urban	0.184^**^	0.235^***^	0.503^***^	0.120^*^	0.254^***^
	(0.063)	(0.058)	(0.071)	(0.063)	(0.058)
Media type (tradition = 0)				0.509^***^	0.041
				(0.066)	(0.059)
Constant	2.334^***^	−0.791^*^	0.864	0.649^**^	0.076
	(0.326)	(0.313)	(0.789)	(0.280)	(0.275)
Observations	8520	8520	8520	8520	8520
Pseudo-R^2^	0.037	0.030	0.220	0.037	0.027
Log likelihood	−5187.199	−5545.856	−3640.407	−5187.765	−5562.692

* *p < 0.05*,

** *p < 0.01*,

*** *p < 0.001*.

*Number (1)–(5) in the table means the number of regression models. Coefficients (log odds) from logit models are presented. The numbers in parentheses are standard errors. All regressions control for province fixed effects; self-reported health statues; and SC-RFC, SC-SV, and SC-NV. Their estimates are omitted here but are available upon request*.

Model 2 in Table 4 shows that education level, ethnic identity, and residential type were significantly associated with the probability of learning of newly known transmission routes. We found that respondents with higher education levels, urban residents, and ethnic Han-Chinese were more likely to know of the updated routes compared with respondents with lower education levels, rural residents, and ethnic minorities. This result further supports that information gap in timeliness across groups is significantly large irrespective of the knowledge on the updated transmission route.

### Effect of Online Media on the Information Gap in Timeliness

In this subsection, we further explore the possible mechanisms of media type behind the differences across groups from different demographics. We used process analysis (33, 34) to assess the intermediary effect of online media on the information gap in timeliness between social groups.

Table 4 (model 3) shows that the educational attainment level and residential type significant affected media type of first informed, which was consistent with the findings from early research. Models 4 (Table 4) shows that media type has a significant effect on early-known. Compared with respondents informed by traditional media, respondents informed by online media showed a 66.36% increase in the odds of early-known (e^0.509^-1). The intermediary effect of online media was also obvious when we compare the two model 1 and model 4. When we included media type in model 4, the coefficient for education level and residential type reduced obviously. This suggests that respondents with different education level and residential type choose different media types which lead some of them get the pandemic information earlier than others. In the other words, part of the information gap in timeliness between education level and residential type groups is attributed to differences in media type.

The coefficient of media type is not significant in terms of whether respondents know of the updated transmission routes (See model 5 in Table 4). This may be because online media helps people get information fast but does not help them distinguish whether the information is accurate, because of the circulation of rumors and fake news (35–37). Even if respondents knew of the new transmission routes, they did not choose them because these routes of transmission had not been confirmed.

While the power of the intermediary effect test with two-step or three-step process analysis is relatively weak (38, 39). To test the effect more rigorously, we present Sobel (40) test and Bootstrap (41) intermediary effect test for media type in Table 5. Both Sobel test and Bootstrap test show that the intermediary effect of media type between age and Early-known, education and Early-known, residential type and Early-known is statistically significant. Because of the higher probability of being informed by online media, younger, high educated and urban residents get higher odds of received information about the outbreak earlier than others. However, the intermediary effect of media type between all variables and Newly-known are not statistically significant, which is consistent with the result from process analysis. The results show that online media help people get the outbreak information earlier, but can't help them know of the updated transmission routes. We think the reason maybe is the rumors and fake news in the online media make them hard to distinguish whether the updated information is accurate.

**Table 5:** The results of Sobel and Bootstrap intermediary effect test for media type.

		**Early-known**		**Newly-known**	
**Test methods**	**Variables**	**Indirect effect**	**Proportion of total effect that is mediated**	**Indirect effect**	**Proportion of total effect that is mediated**
Sobel	Age	−0.001^***^	18.80%	0.001	−44.7%
	Education	0.011^***^	22.90%	0.001	1.7%
	Residential type	0.001*	9.70%	0.001	0.5%
	Ethnic minority	0.003	12.80%	0.001	−0.8%
Bootstrap	Age	−0.001^***^	18.80%	−0.001^**^	−44.7%
	Education	0.011^***^	22.90%	0.001	1.7%
	Residential type	0.001^+^	9.70%	0.001	0.5%
	Ethnic minority	0.003	12.80%	0.001	−0.8%

+ *p < 0.10, p < 0.05*,

** *p < 0.01*,

*** *p < 0.001*.

*To estimate the total effect and make the result easy to understood, the categorical variables education, residential type and ethnicity are treated as continuous variable*.

## Concluding Remark

COVID-19 has challenged the global public health system in terms of developing effective control strategies to stop its spread. Effective health communication helps battle misinformation and reduce panic and its consequent health risks as well as plays a key role during public health emergencies. During such emergencies, less educated individuals, indigenous individuals, older individuals, and rural and remote-region residents do not get accurate and timely health emergency information (14, 42). This kind of communication inequality is quite common, which causes marginalized social groups to be at a higher risk than estimated and to be less likely to follow recommended behaviors (13, 15, 16). Baced on that knowledge, researchers argue that developing countries with weak health systems and regional, cultural, linguistic and ethnic diversity should pay more attention to the role of effective communication, without leaving anyone behind when communicating crisis and risk to the population to address the COVID-19 pandemic (43).

This research takes a quantitative approach toward examining possible communication inequalities among people in rural and remote regions. Differing from the existing literature, we evidence that the marginalized groups obtained accurate health information, which helped them adopt appropriate protective measures. This improved the effectiveness of government policy for transmission control, as evidenced by the current decrease in COVID-19 cases (5). However, the research also suggests that knowledge updates among disadvantage groups such as order, less educated and rural residents remain comparatively slow. Online media that are more accessible to urban, younger and educated residents may the disadvantage groups acquire information at a faster rate than others.

The policy implications from this research for China and other countries are two-fold: (1) it is critical for governments to broaden the channels of information dissemination, particular improving the use of online media, (2) there is a requirement for continuous investment by the government toward providing disadvantage people in remote regions with accessibility to up to date information and knowledge. As the first pandemic of the social media age, social media communication played a significant role in the pandemic of COVID-19. The extensive global penetration of social media provided a fertile ground for the spread of information, misinformation, and fake news (44). On the other hand, the COVID-19 crisis exacerbated the already existed “digital inequality” dramatically, worsen it within the population (45). How to face the rise of “digital inequality” as well as solve the problem of “infodemic”? Based on the results of this research and practical implications from other countries, we believe that mobilizing local governments and social organizations is an effective strategy to reduce digital inequality. Both of them could serve as avenues for risk communication when social media is not accessible. In addition to this, the government should expand access to social media, make more people have the opportunity to access to internet. Research shows that health policy influences search behavior on the internet (46), and we believe that the government should leverage these the risk communication strategies in the social media age. To prevent information vacuums that get filled in by unreliable lay advice and rumors, the government should use both traditional broadcast media channels (media briefings and press releases) and more direct channels to provide scientific and reliable information to combat misinformation and disinformation (47).

Finally, differing from existing COVID-19 study done by Wang and others (34), we covered a wide geographic coverage to improve the sample representative by assigning 7 universities to particularly carry out survey in their province. But there is still limitation of this study. Restricted by the time frame and the unusually strict quarantine measures at the time of our data collection, the data collection process of this study was not able to follow a random selection process, similar as other COVID-19 study (27, 48). Our results are, therefore, inevitably subject to potential biases due to the sampling procedure. China has approximately 932 million mobile Internet users (67 percent of population)^5^, yet more than 360 million, who are more likely to be less educated and live in rural areas, do not have access to smart phone. Because our survey is conducted through a mobile app platform, such groups are inherently underrepresented in our data. This selection bias may lead to underestimation of the inequality in knowledge dissemination. It would prove productive to conduct retrospective studies with a more representative sample to compare against our findings. We leave that effort to future research.

## Data Availability Statement

The raw data supporting the conclusions of this article will be made available by the authors, without undue reservation.

## Ethics Statement

The studies involving human participants were reviewed and approved by Institutional committee of Yunnan University, School of Ethnology and Sociology. Written informed consent for participation was not required for this study in accordance with the national legislation and the institutional requirements.

## Author Contributions

WZ, LH, JH, and MH: conceptualization. JH, LH, WZ, TW, JP, DC, HG, JL, KT, XN, and MH: methodology, investigation, and writing—review and editing. JH, LH, and WZ: formal analysis and writing—original draft preparation. JH and MH: supervision, project administration, and funding acquisition.

## Funding

The research received financial support from various sources: WZ received financial support from the China Postdoctoral Science Foundation (No. 2019M663590); MH, from the National Social Sciences Foundation of China (No. 16ZDA151); JH, from the Ministry of Education of People's Republic of China (Project No. 16JJD850015).

## Conflict of Interest

The authors declare that the research was conducted in the absence of any commercial or financial relationships that could be construed as a potential conflict of interest.

## Publisher's Note

All claims expressed in this article are solely those of the authors and do not necessarily represent those of their affiliated organizations, or those of the publisher, the editors and the reviewers. Any product that may be evaluated in this article, or claim that may be made by its manufacturer, is not guaranteed or endorsed by the publisher.

## Footnotes

^1^See http://data.stats.gov.cn/easyquery.htm?cn=E0103.

^2^According to previous research for government behavior and area development (28–30), we believe that the main difference of risk communication come from the county level, especially when it was led by government. To increase geographical coverage for the selection of sufficient districts and counties, while at the same time reducing the workload and costs of the cooperating agencies, we selected ten counties from all counties in every province and every county selected two villages to conduct our survey.

^3^See https://www.thepaper.cn/newsDetail_forward_5734999.

^4^See https://www.who.int/dg/speeches/detail/munich-security-conference (access on 1 March, 2020).

^5^Statistical Report on Internet Development in China (September 2020), released by China Internet Network Information Center, See https://www.cnnic.net.cn/hlwfzyj/hlwxzbg/hlwtjbg/202009/t20200929_71257.htm.
