# Supplementary material for: Accuracy and Timeliness of Knowledge Dissemination on COVID-19 Among People in Rural and Remote Regions of China at the Early Stage of Outbreak
Source: Front Public Health. 2022 Jan 11;9:554038. doi: 10.3389/fpubh.2021.554038 (PMC8787119; doi:10.3389/fpubh.2021.554038)
Supplement: Supplementary file 1 [file Table_1.docx]

Appendix Table 1. Descriptive statistics of survey data

|  |  | **Frequency** | **Percentage** |
| --- | --- | --- | --- |
| Age group | 10-20 | 1,364 | 16.01 |
|  | 21-35 | 3,973 | 46.63 |
|  | 36-50 | 2,452 | 28.78 |
|  | 51-65 | 674 | 7.91 |
|  | >65 | 57 | 0.67 |
| Sex | female | 5,698 | 66.88 |
|  | male | 2,822 | 33.12 |
| Education background | Primary school | 168 | 1.97 |
|  | Middle school | 593 | 6.96 |
|  | High school | 1,038 | 12.18 |
|  | College | 4,915 | 57.69 |
|  | Post graduate | 1,806 | 21.2 |
| Ethnic identity | Ethnic minority | 3,506 | 41.15 |
|  | Ethnic Han | 5,014 | 58.85 |
| Rural-urban residential type | rural | 2,516 | 29.53 |
|  | suburb | 947 | 11.12 |
|  | urban | 5,057 | 59.35 |
| Province | inner Mongolia | 669 | 7.85 |
|  | Jilin | 882 | 10.35 |
|  | Heilongjiang | 493 | 5.79 |
|  | Guangxi | 716 | 8.4 |
|  | Yunnan | 4,595 | 53.93 |
|  | Tibet | 444 | 5.21 |
|  | Xinjiang | 721 | 8.46 |
