# Supplementary material for: Accuracy and Timeliness of Knowledge Dissemination on COVID-19 Among People in Rural and Remote Regions of China at the Early Stage of Outbreak
Source: Front Public Health. 2022 Jan 11;9:554038. doi: 10.3389/fpubh.2021.554038 (PMC8787119; doi:10.3389/fpubh.2021.554038)
Supplement: Supplementary file 2 [file Table_2.docx]

Appendix Table 2. Selection bias of our research data

| Provinces | Population and ethnic minorities | | | Urbanization rate | | Average age | |
| --- | --- | --- | --- | --- | --- | --- | --- |
|  | Sample | Official statistics  （2019） | The number of ethnic minorities | Sample | Official statistics  （2019） | Sample | Official statistics  （2015） |
| Inner Mongolia | 669 | 25396000 | 4 | 49.8 | 63.4 | 32 | 39 |
| Jilin | 882 | 26907000 | 5 | 70.0 | 58.3 | 35 | 40 |
| Heilongjiang | 493 | 37513000 | 10 | 76.5 | 60.9 | 30 | 40 |
| Guangxi | 716 | 49600000 | 7 | 48.2 | 51.1 | 29 | 37 |
| Yunnan | 4595 | 48583000 | 25 | 59.4 | 48.9 | 33 | 37 |
| Tibet | 444 | 3506000 | 3 | 19.6 | 31.5 | 26 | 34 |
| Xinjiang | 721 | 25232000 | 8 | 64.6 | 51.9 | 32 | 37 |
